# Metabolome and Transcriptome Profiling Reveals the Function of MdSYP121 in the Apple Response to *Botryosphaeria dothidea*

**DOI:** 10.3390/ijms242216242

**Published:** 2023-11-13

**Authors:** Jiahu Zhang, Sen Wang, Haibo Wang, Ping He, Yuansheng Chang, Wenyan Zheng, Xiao Tang, Linguang Li, Chen Wang, Xiaowen He

**Affiliations:** 1 Shandong Institute of Pomology, Tai’an 271000, China; zjh20210307@163.com (J.Z.); gsswangsen@shandong.cn (S.W.); gsswanghaibo@shandong.cn (H.W.); gssheping@shandong.cn (P.H.); changyuansheng@shandong.cn (Y.C.); zhengwy202210@163.com (W.Z.); llg6536@163.com (L.L.); 2 College of Life Sciences, Shandong Agricultural University, Tai’an 271018, China; t13210119481@126.com (X.T.); cwang@sdau.edu.cn (C.W.)

**Keywords:** apple, *MdSYP121*, *Botryosphaeria dothidea*, metabolome, transcriptome, sugar

## Abstract

The vesicular transport system is important for substance transport in plants. In recent years, the regulatory relationship between the vesicular transport system and plant disease resistance has received widespread attention; however, the underlying mechanism remains unclear. MdSYP121 is a key protein in the vesicular transport system. The overexpression of *MdSYP121* decreased the *B. dothidea* resistance of apple, while silencing *MdSYP121* resulted in the opposite phenotype. A metabolome and transcriptome dataset analysis showed that *MdSYP121* regulated apple disease resistance by significantly affecting sugar metabolism. HPLC results showed that the levels of many soluble sugars were significantly higher in the *MdSYP121*-OE calli. Furthermore, the expression levels of genes related to sugar transport were significantly higher in the *MdSYP121*-OE calli after *B. dothidea* inoculation. In addition, the relationships between the *MdSYP121* expression level, the soluble sugar content, and apple resistance to *B. dothidea* were verified in an F1 population derived from a cross between ‘Golden Delicious’ and ‘Fuji Nagafu No. 2’. In conclusion, these results suggested that *MdSYP121* negatively regulated apple resistance to *B. dothidea* by influencing the soluble sugar content. These technologies and methods allow us to investigate the molecular mechanism of the vesicular transport system regulating apple resistance to *B. dothidea*.

## 1. Introduction

Apple (*Malus domestica* Borkh.) is one of the most widely produced and economically important fruits in the world [1]. In recent years, due to its composition of a variety of nutrients with different biological activities, including polyphenols, vitamins, minerals, lipids, proteins/peptides, and carbohydrates, and the fact that it is convenient to consume, apple has been among the most widely consumed fruits globally [2,3]. In China, apple cultivation is threatened by apple ring rot caused by *Botryosphaeria dothidea*. *B. dothidea* is one of the most common and destructive fungal pathogens, causing serious fruit rot, damaging tree branches, and resulting in tree weakness or the death of the whole plant [4,5]. On the fruit, *B. dothidea* causes slightly sunken lesions with alternating tan and brown rings, seriously decreasing the sales value of the fruit and leading to enormous economic losses in apple production [4]. With the large-scale cultivation of Fuji and other high-quality varieties that are susceptible to *B. dothidea*, the frequency and severity of apple ring rot have significantly increased [6]. Therefore, systematically understanding apple’s defense mechanism is of great significance for the effective prevention and control of the damage caused by *B. dothidea*, to improve the storage level of apple fruits and to promote the healthy development of the Chinese apple industry.

Plants have developed complex defense mechanisms in response to pathogen infection over their long-term evolution. Many studies have shown that plants transport resistance-related substances (such as callose, phytoalexin, and polypeptides) to apoplasts or various organelles to respond to pathogen attacks through their unique intimal system and vesicular trafficking pathway [7,8,9]. In plants, the vesicular trafficking pathway includes the formation and transport of vesicles, and the recognition and fusion between vesicles and target membranes, which are the key steps of vesicle transport [10]. Soluble *N*-ethylmaleimide-sensitive factor attachment protein receptor (SNARE) is a key regulator of vesicle trafficking and mediates the fusion between vesicles and target membranes [11]. SNARE proteins also play specialized roles in a variety of biological processes, such as in maintaining cellular homeostasis and facilitating plant nutrition, growth, and immunity [12,13]. The first identified SNARE that was shown to be required for plant immunity was the *Arabidopsis* PM-residing AtSYP121 (also called AtPEN1) Qa-SNARE, whose barley ortholog is HvROR2 (required for mlo-specified disease resistance 2) [14]. SYP121 plays complex and different roles in the response to pathogens in different species. Under *Plectosphaerella cucumerina* infection, cucumber SYP121 can interact with the ubiquitin ligase ATL31 and regulate callose accumulation as a basic defense response [15]. AtSYP121 in epidermal cells interacts with EXO70B2 and controls callose formation in response to powdery mildew [16]. However, in potato, *StSYR1* (*AtSYP121* homologous gene) plays a different role in the plant defense response. Under *Phytophthora infestans* infection, *StSYR1*-silenced lines accumulated more salicylic acid and exhibited higher resistance to late blight [17]. Our previous studies have shown that apple SYP121, *MdSYP121*, negatively regulates the resistance to *B. dothidea* [18]. However, the regulatory mechanism of *MdSYP121* in response to *B. dothidea* is still unclear. Further analysis of the molecular mechanism by which *MdSYP121* regulates the apple response to *B. dothidea* is of great significance for guiding apple genetic breeding and cultivation, and improving the theoretical knowledge of the vesicular trafficking and metabolic substances involved in disease resistance.

In plants, sugar is involved in many metabolic and transport pathways, and is essential for plant growth, development, and response to abiotic and biotic stresses [19,20]. Relevant studies have shown that sugars are also nutrient sources for plant pathogens. For example, high sugar content in the medium was shown to promote *Rhizoctonia solani* hyphal growth [21]. In most cases, plant pathogens cannot directly use starch and sucrose for their growth, as these must be decomposed to monosaccharides such as glucose and fructose [22,23]. Previous research has found that the metabolism of sugars is accelerated in galls generated following inoculation with *Meloidogyne incognita*, and the levels of starch, sucrose, and glucose showed obvious accumulation [24]. It was recently reported that plant-specific membrane trafficking mechanisms might be involved in gall formation [25]. Zhang et al. [26] found that after powdery mildew infection, the levels of glucose and fructose in the resistant Kentucky bluegrass variety ‘BlackJack’ decreased, while the levels in the susceptible variety ‘EverGlade’ increased. These studies indicated that nutrient competition is a key mechanism involved in the interaction between pathogens and hosts.

In this study, metabolome and transcriptome datasets of *MdSYP121*-OE calli and Vec lines before and after inoculation with *B. dothidea* and a series of genetic and biochemical experiments defined the relationship between *MdSYP121*, soluble sugars, and the apple response to *B. dothidea*. These technologies and methods allow us to investigate the molecular mechanism of the vesicular transport system regulating apple resistance to *B. dothidea* and provide reference materials for the breeding of new high-quality and high-resistance apple varieties.

## 2. Results

### 2.1. The Role of MdSYP121 in Apple Resistance to B. dothidea

**Figure 1:**
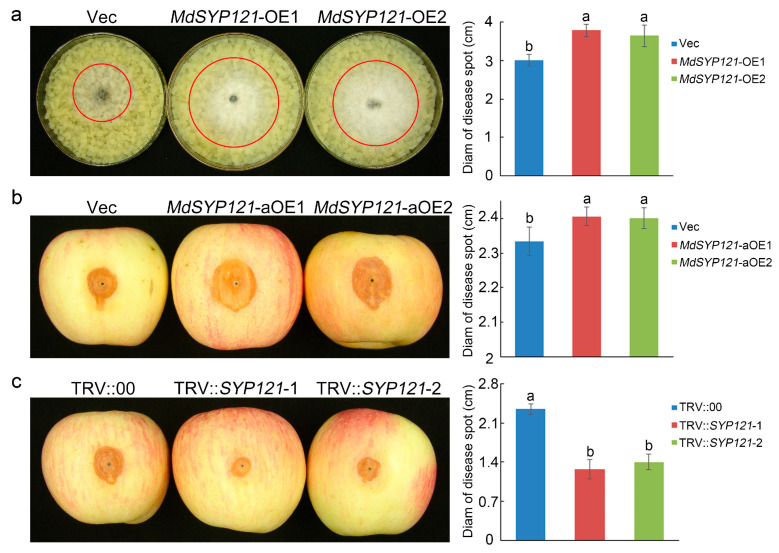
Phenotype and disease lesions of transgenic apple calli and transient transgenic apple fruit infected with *B. dothidea*: (**a**) phenotype and average diameters of lesions of Vec and *MdSYP121*-overexpressing transgenic apple calli infected with *B. dothidea*; (**b**) phenotype and average diameters of lesions of Vec apple fruit and apple fruit transiently overexpressing *MdSYP121* infected with *B. dothidea*; (**c**) phenotype and average diameters of lesions of Vec apple fruit and apple fruit with transiently silenced *MdSYP121* infected with *B. dothidea*. Error bars indicate the mean ± standard error of three independent experiments (*n* = 9). Different letters indicate significant differences (*p* < 0.01) based on Tukey’s HSD test.

According to our previous research, *MdSYP121* played negative roles in apple resistance to *B. dothidea* [18]. *MdSYP121*-OE transgenic apple calli were generated and confirmed using RT–qPCR and Western blotting (Figure S1). As shown in Figure 1a, the *MdSYP121*-OE calli had higher sensitivity to *B. dothidea* than the Vec lines. At 4 days post inoculation, the fungal extension of *B. dothidea* on *MdSYP121*-OE apple calli was significantly greater than that on Vec apple calli (Figure 1a). Compared with the Vec lines, the spot extension areas of the *MdSYP121*-OE lines indicated a nearly 1.3-fold increase in fungal growth (Figure 1a). The transient overexpression of *MdSYP121* or silencing of *MdSYP121* via virus-induced gene silencing (VIGS) assays in apple fruit were used to further analyze the effect of *MdSYP121* on apple disease resistance. Four days after *B. dothidea* inoculation, consistent with the results for apple calli, the lesions on the apple fruit overexpressing *MdSYP121* were significantly larger than those on the control fruit, while the silencing of *MdSYP121* resulted in significantly smaller lesions than those on the control fruit (Figure 1b,c). These results verified that *MdSYP121* negatively regulated apple resistance to *B. dothidea*.

### 2.2. Metabolome Profiling of MdSYP121-OE Calli Responding to B. dothidea

**Figure 2:**
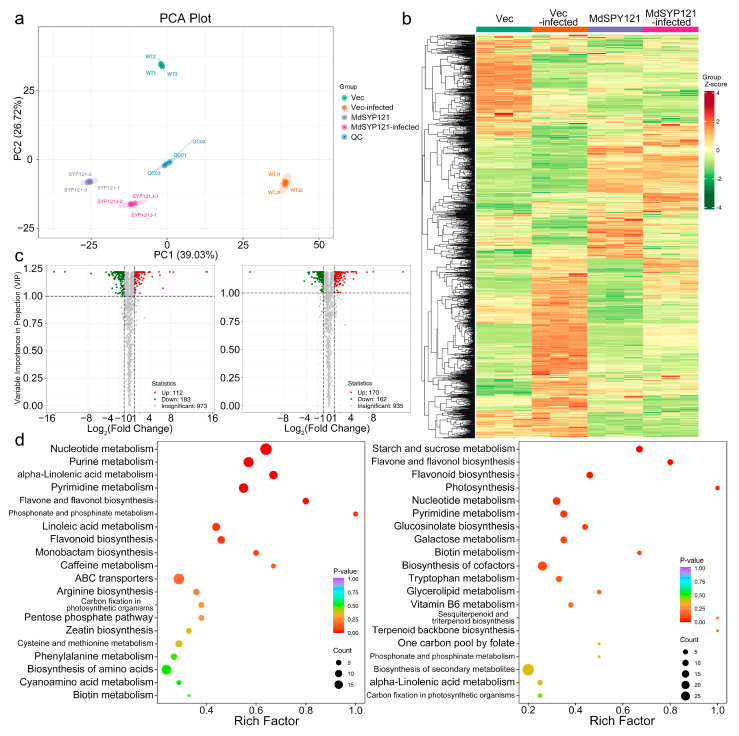
Differentially accumulated metabolites (DAMs) of Vec and *MdSYP121*-OE calli before and after *B. dothidea* inoculation. (**a**) PCA plots of metabolism identified by LC–MS/MS of Vec and *MdSYP121*-OE calli before and after *B. dothidea* inoculation. (**b**) Cluster analysis heatmap of Vec and *MdSYP121*-OE calli before and after *B. dothidea* inoculation. (**c**) Volcano plot analysis of Vec and *MdSYP121*-OE calli before and after *B. dothidea* inoculation. Left: Vec/MdSYP121. Right: Vec-infected/MdSYP121-infected. (**d**) KEGG functional analysis of Vec and *MdSYP121*-OE calli before and after *B. dothidea* inoculation. Left: Vec/MdSYP121. Right: Vec-infected/MdSYP121-infected.

To further investigate the mechanism of action of *MdSYP121* in the apple response to *B. dothidea*, metabolomics analysis was performed. Metabolites were extracted from the calli of the *MdSYP121*-OE and Vec lines uninfected or infected with *B. dothidea* for 4 days. PCA showed a significant difference among the different treatments and calli, and the three biological replicates of each treatment were clustered together (Figure 2a). The metabolome data were analyzed using UPLC–MS/MS. A total of 1268 metabolites, which belonged to 11 classes, were detected (Figure 2b).

A total of 295 differentially accumulated metabolites (DAMs) with |log2FC| ≥ 1 and VIP ≥ 0.9 between the Vec calli (Vec) and *MdSYP121*-OE calli (MdSYP121) were detected, of which 112 were upregulated and 183 were downregulated (Table S1 and Figure 2c). In contrast, there were 332 DAMs, more than in Vec/MdSYP121, in the Vec calli inoculated with *B. dothidea* (Vec-infected) compared with the *MdSYP121*-OE calli infected with *B. dothidea* (MdSYP121-infected), among which 170 were upregulated and 162 were downregulated (Table S2 and Figure 2c).

A KEGG enrichment analysis was performed to explore the functions of the metabolites that were altered in apple calli after *B. dothidea* infection. The results indicated that most DAMs in the Vec/MdSYP121 comparison group were significantly enriched in many pathways, such as nucleotide metabolism (ko01232), purine metabolism (ko00230), and alpha-linolenic acid metabolism (ko00592) (Figure 2d and Table S3). However, the DAMs in the Vec-infected/MdSYP121-infected comparison group were significantly enriched in starch and sucrose metabolism (ko00500), flavone and flavonol biosynthesis (ko00944), and flavonoid biosynthesis (ko00941) (Figure 2d and Table S4). These findings indicated that the metabolic pathway changed significantly in the calli after inoculation with *B. dothidea. MdSYP121* might regulate the apple response to *B. dothidea* by affecting sugar metabolism.

To study the trend of the relative levels of metabolites in different groups, the relative levels of all DAMs identified by the screening criteria in all comparison groups were subjected to UV scaling, and then a K-means clustering analysis was conducted. The results showed that saccharides such as 2-dehydro-3-deoxy-L-arabinonate, galactinol, D-fructose 6-phosphate, D-sucrose, and D-glucose 6-phosphate were closely correlated with the apple response to *B. dothidea*. Compared with Vec-infected calli, these metabolites were significantly enriched in *MdSYP121*-OE calli infected with *B. dothidea* (Figure S2 and Table S5).

Taken together, these results demonstrated that *MdSYP121* had a profound impact on apple carbohydrate metabolism and then influenced the apple response to *B. dothidea*.

### 2.3. Transcriptome Analysis of MdSYP121-OE Calli Responding to B. dothidea

**Figure 3:**
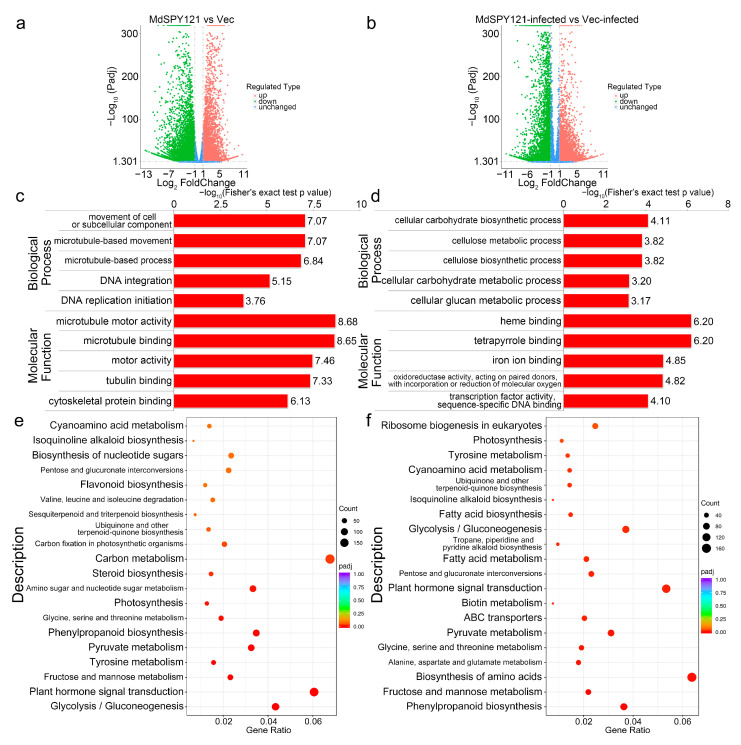
Differentially expressed genes (DEGs) of Vec and *MdSYP121*-OE calli before and after *B. dothidea* inoculation. (**a**) Volcano map of DEGs in Vec/MdSYP121. (**b**) Volcano map of DEGs in Vec-infected/MdSYP121-infected. (**c**) GO terms of DEGs in Vec/MdSYP121. (**d**) GO terms of DEGs in Vec-infected/MdSYP121-infected. (**e**) KEGG pathway analysis of DEGs in Vec/MdSYP121. (**f**) KEGG pathway analysis of DEGs in Vec-infected/MdSYP121-infected.

To further investigate the molecular mechanism by which *MdSYP121* participates in apple resistance to *B. dothidea*, a transcriptomic analysis of Vec and *MdSYP121*-OE calli inoculated or uninoculated with *B. dothidea* was performed. After trimming the adaptor sequences and removing low-quality reads, 29.65 Gb of clean reads was generated. Adjusted *p* value ≤ 0.05 and |log2-fold fold change| ≥ 1 were set as the thresholds to determine the significance of the difference in gene expression between samples. Without *B. dothidea* infection, 13,791 DEGs, including 5870 upregulated and 7921 downregulated genes, were identified between Vec and *MdSYP121*-OE calli, whereas 12,810 DEGs, including 6867 upregulated and 5943 downregulated genes, were identified between Vec and *MdSYP121*-OE calli after *B. dothidea* inoculation (Figure 3a,b, and Tables S6 and S7).

To further understand the function of the DEGs and the related biological processes in which they participate, GO enrichment analyses were performed. The GO analysis classified the DEGs in Vec/MdSYP121 and Vec-infected/MdSYP121-infected calli into different biological processes and molecular functions. Significantly, within the biological process category, the main enriched GO terms in Vec/MdSYP121 were movement of cell or subcellular component (GO:0006928), microtubule-based movement (GO:0007018), and microtubule-based process (GO:0007017), while those in Vec-infected/MdSYP121-infected were cellular carbohydrate biosynthetic process (GO:0034637), cellulose metabolic process (GO:0030243), and cellulose biosynthetic process (GO:0030244). Within the molecular function category, the main enriched GO terms in Vec/MdSYP121 were microtubule motor activity (GO:0003777), microtubule binding (GO:0008017), and motor activity (GO:0003774), while those in Vec-infected/MdSYP121-infected were heme binding (GO:0020037), tetrapyrrole binding (GO:0046906), and iron ion binding (GO:0005506) (Figure 3c,d, and Tables S8 and S9).

To better analyze the function of these DEGs in apple resistance to *B. dothidea*, we performed a KEGG analysis of the DEGs. The pathway enrichment analysis of the Vec/MdSYP121 DEGs showed that glycolysis/gluconeogenesis (mdm00010), plant hormone signal transduction (mdm04075), fructose and mannose metabolism (mdm00051), tyrosine metabolism (mdm00350), and pyruvate metabolism (mdm00620) were significantly enriched (Figure 3e and Table S10). However, in the Vec-infected/MdSYP121-infected group, the genes mostly enriched the phenylpropanoid biosynthesis (mdm00940), fructose and mannose metabolism (mdm00051), biosynthesis of amino acids (mdm01230), alanine, aspartate, and glutamate metabolism (mdm00250), and glycine, serine, and threonine metabolism (mdm00260) pathways (Figure 3f and Table S11).

Taken together, the transcriptome analysis results consistently showed that apple resistance to *B. dothidea* was significantly related to carbohydrate metabolism.

### 2.4. MdSYP121 Influenced the Soluble Sugar Content and the Expression Level of Sugar Transport Genes

**Figure 4:**
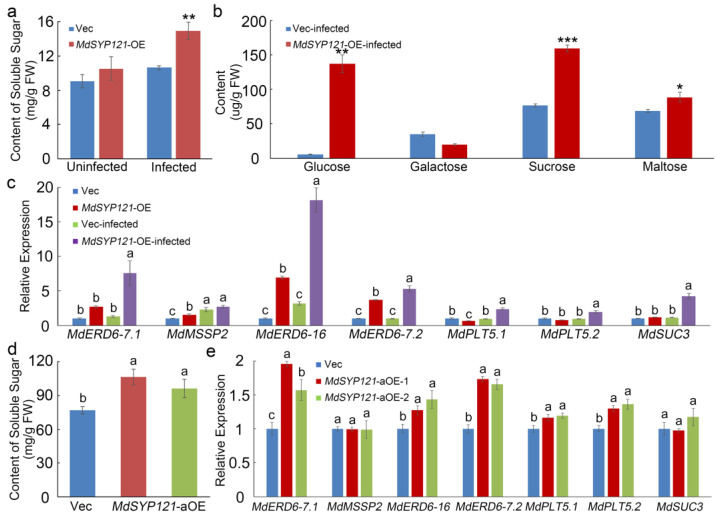
*MdSYP121* had a positive effect on sugar metabolism. (**a**) The soluble sugar content in *MdSYP121*-OE calli and Vec calli before and after *B. dothidea* inoculation; the data are the mean ± standard error of three independent experiments (*n* = 9). ** *p* < 0.01 based on Tukey’s HSD test. (**b**) HPLC analysis of glucose, galactose, sucrose, and maltose in *MdSYP121*-OE calli and Vec calli after *B. dothidea* inoculation; the error bars indicate the mean ± standard error of three independent experiments (*n* = 9). * *p* < 0.05; ** *p* < 0.01; *** *p* < 0.001. (**c**) The expression levels of related sugar transporter genes in *MdSYP121*-OE calli and Vec calli before and after *B. dothidea* inoculation. (**d**) The soluble sugar content in apple fruit transiently overexpressing *MdSYP121*. (**e**) The expression levels of related sugar transporter genes in apple fruit transiently overexpressing *MdSYP121*. Error bars in (**c**,**d**,**e**) indicate the mean ± standard error of three independent experiments (*n* = 9). Different letters indicate significant differences (*p* < 0.01) based on Tukey’s HSD test.

A combined analysis of the metabolome and transcriptome showed that *MdSYP121* had a significant effect on starch and sucrose metabolism. Therefore, the soluble sugar content in both *MdSYP121*-OE calli and Vec calli uninfected or infected with *B. dothidea* was measured. The soluble sugar content had no obvious difference in the *MdSYP121*-OE calli and in the Vec calli (Figure 4a). After *B. dothidea* inoculation, the soluble sugar content was significantly higher in the *MdSYP121*-OE calli (Figure 4a). HPLC analysis results indicated that the levels of glucose, sucrose, and maltose were significantly higher in the *MdSYP121*-OE calli than in the Vec calli after inoculation with *B. dothidea*. However, in contrast to that of glucose, sucrose and maltose, the accumulation of galactose was lower in the *MdSYP121*-OE calli than in the Vec calli (Figure 4b).

In addition, RT–qPCR was performed to measure the expression levels of genes related to starch and sucrose metabolism pathways in different calli. Six sugar transporter genes (*MdERD6-7.1*, *MdMSSP2*, *MdERD6-16*, *MdERD6-7.2*, *MdERDPLT5.1*, *MdERDPLT5.2*, and *MdSUC3*) were selected from the transcriptome dataset. The selected genes showed the same increasing expression profile and higher expression level in the *MdSYP121*-OE calli than in the Vec calli (Figure 4c). Furthermore, the expression levels of these genes were even higher in *MdSYP121*-OE calli after *B. dothidea* inoculation (Figure 4c). To further verify this result, the soluble sugar content and related gene expression in apple fruit transiently overexpressing *MdSYP121* were measured before and after *B. dothidea* inoculation. The results suggested that the soluble sugar content was significantly higher than that in the control. The RT–qPCR results also showed that the expression levels of the sugar transporter genes *MdERD6-7.1*, *MdERD6-16*, *MdERD6-7.2*, *MdERDPLT5.1*, *MdERDPLT5.2*, and *MdSUC3* were increased, while there was no significant change in the expression levels of *MdMSSP2* in apple fruit transiently overexpressing *MdSYP121* (Figure 4d,e).

**Figure 5:**
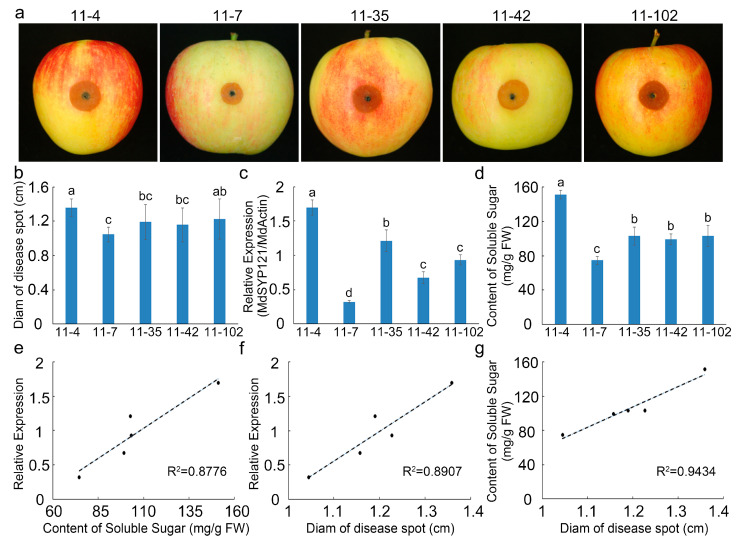
Phenotypes of apple in response to *B. dothidea* in the F1 population derived from a cross between ‘Golden Delicious’ and ‘Fuji Nagafu No. 2’. (**a**) Phenotypes of the fruit of five individual lines of apple (11-4, 11-7, 11-35, 11-42, and 11-102) after *B. dothidea* inoculation. (**b**) Diameter of disease spots on apple fruit of the five individual lines (11-4, 11-7, 11-35, 11-42, and 11-102) after *B. dothidea* inoculation; error bars indicate the mean ± standard error of three independent experiments (*n* = 6). Different letters indicate significant differences (*p* < 0.01) based on Tukey’s HSD test. (**c**) Relative expression of *MdSYP121* in the apple fruit of the five individual lines (11-4, 11-7, 11-35, 11-42, and 11-102) after *B. dothidea* inoculation; error bars indicate the mean ± standard error of three independent experiments (*n* = 6). Different letters indicate significant differences (*p* < 0.01) based on Tukey’s HSD test. (**d**) The soluble sugar content in the apple fruit of the five individual lines (11-4, 11-7, 11-35, 11-42, 11-102) after *B. dothidea* inoculation; error bars indicate the mean ± standard error of three independent experiments (*n* = 6). Different letters indicate significant differences (*p* < 0.01) based on Tukey’s HSD test. (**e**) Correlation between *MdSYP121* expression levels and soluble sugar content. (**f**) Correlation between *MdSYP121* expression levels and disease spot diameter. (**g**) Correlation between soluble sugar content and disease spot diameter.

In our previous research, to identify new apple genotypes with high quality and disease resistance, we constructed an F1 population derived from a cross between ‘Golden Delicious’ and ‘Fuji Nagafu No. 2’. To further elucidate the mechanism by which *MdSYP121* decreased apple resistance to *B. dothidea*, the relationships between the expression level of *MdSYP121*, the soluble sugar content, and apple resistance to *B. dothidea* were examined. Five individual lines (11-4, 11-7, 11-35, 11-42, and 11-102) were randomly selected. After inoculation with *B. dothidea* for 4 days, the disease lesion area and the diameter of the disease spot in the 11-4 line were the largest, those in the 11-7 line were the smallest, and those in 11-35, 11-42, and 11-102 were similar and showed intermediate values (Figure 5a,b). The RT–qPCR results showed that the *MdSYP121* expression level was highest in the 11-4 line and lowest in the 11-7 line (Figure 5c). Additionally, the soluble sugar content was measured in the five randomly selected samples. The 11-4 line had the maximum soluble sugar content, the 11-7 line had the minimum soluble sugar content, and the other three lines showed no significant difference (Figure 5d). The results showed that a high percentage of the soluble sugar was easily detectable in the highly susceptible strain, and *B. dothidea* was more harmful to the highly susceptible progeny (Figure 5d). The relationships between *MdSYP121* expression levels, soluble sugar content, and disease spot diameter showed high correlation coefficients (Figure 5e–g).

## 3. Discussion

Apple production in China accounts for more than 50% of the global production. Apple cultivation is a pillar industry for increasing agricultural income, but the apple industry is vulnerable to many diseases. Apple ring rot is one of the main diseases in the major apple production region and seriously affects the healthy development of the apple industry. Our previous studies have shown that a Qa-SNARE group protein, MdSYP121, plays a negative role in apple resistance to *B. dothidea* [18]. In the present study, metabolome and transcriptome analyses were performed on *MdSYP121*-OE calli and Vec calli infected and uninfected with *B. dothidea*. The results of a series of genomic, genetic, and transgenic experiments suggest that *MdSYP121* is related to the accumulation of soluble sugars in apple, and that the negative regulation of *MdSYP121* in *B. dothidea* can be attributed to the increase in the soluble sugar content.

In plants, protein secretion appears to be an important, and possibly terminal, step mediating active resistance to pathogen infection. SNARE proteins function as mediators of fusion between vesicular and target membranes [27,28,29]. In *Arabidopsis*, VAMP727-SYP22 SNARE complexes negatively regulate plant defense against root-knot nematode (RKN), a plant pathogen that causes severe growth defects and yield loss [30]. SYP132 is a member of the SNARE family and is essential for post penetration plant immunity [31,32]. In *Arabidopsis*, *SYP132* is involved in the response to *Pseudomonas syringae* pv. tomato DC3000 (*Pst* DC3000) infection through the stomatal route [33]. The SNAREs (or syntaxins) SYP121 and SYP132, two homologous SNAREs, share over 60% sequence similarity and have overlapping as well as distinct functions [11]. AtSYP121 was the first identified SNARE shown to be required for plant immunity [14]. AtSYP121 facilitates focal secretory trafficking for the penetration response to the powdery mildew fungus [34]. OsSYP121 was reported to be required for rice resistance to the rice blast fungus *Magnaporthe oryzae* [16]. Our study indicated that *MdSYP121* played a negative regulatory role in apple resistance to *B. dothidea*. Overexpressing the *MdSYP121* gene decreased apple calli and fruit tolerance to *B. dothidea* infection. The fungal extension of *B. dothidea* on *MdSYP121*-OE apple calli and fruit was significantly greater than that on the control.

To better investigate the molecular mechanism of action of *MdSYP121* in regulating apple resistance to *B. dothidea*, a metabolome and transcriptome analysis was used to study the change trends of the metabolite content and gene expression levels before and after inoculation. Based on the metabolome and transcriptome analysis results, many sugar metabolites associated with biotic stress were upregulated in the *MdSYP121*-OE lines and were significantly related to the apple response to *B. dothidea*. Plants have evolved complex mechanisms for responding to pathogen infection over the period of their growth and development [35]. Sugars are important molecules that control almost all morphological and physiological processes in plants [36]. The role that sugar transporters play in pathogen susceptibility was predicted 30 years ago [37]. Previous studies have hypothesized that sugar-mediated pathogen resistance can occur via ‘pathogen starvation’ or ‘sugar signaling’ [38]. The *Puccinia striiformis* f. sp. *tritici* (Pst)-induced sugar transporter TaSTP3 is transcriptionally activated by TaWRKY19/61/82 and facilitates wheat susceptibility to stripe rust, possibly through elevated sucrose concentrations [39]. In *Arabidopsis*, the hexose transporter STP13 level balances hexose fluxes to mediate host–pathogen interactions [40]. Early responsive to dehydration (ERD) genes are rapidly induced in response to various biotic stresses in *Arabidopsis* [41]. Biological stress in plants is mainly caused by various pests and pathogens, such as *Bacillus subtilis* and other bacteria, fungi, oomycetes, phytoplasmas, and viruses [42]. It is usually caused by infection and competition. *AtERD15* encodes a small acidic protein and is involved in the response to biological stress [43]. In this study, a metabolome and transcriptome analysis, conducted via HPLC and RT–qPCR, showed that higher levels of sugars and higher expression levels of sugar transporter-related genes were observed in the *MdSYP121*-OE lines than in the Vec lines (Figure 2, Figure 3 and Figure 4). In addition, verification experiments via F1 hybrids infection showed that a high expression level of *MdSYP121* was easily detectable in the highly susceptible strain and was related to a high percentage of soluble sugars and the response to *B. dothidea* (Figure 5). Based on the results, we speculated that *MdSYP121* regulated disease resistance to *B. dothidea* by influencing sugar metabolism.

In this study, using a metabolome and transcriptome analysis, we found that *MdSYP121* had a significant effect on starch and sucrose metabolism. Sugars are important metabolic products of plants and play a vital role in the interaction between plants and pathogens. Genetic transformation and biochemical experiments proved that the overexpression of *MdSYP121* significantly increased the levels of glucose, sucrose, and maltose and the expression of related sugar transporter genes. We speculated that *MdSYP121* may be a regulatory site for balancing fruit sugar content and apple resistance to *B. dothidea*. Our results enrich the knowledge of the molecular mechanism by which the vesicular transport system regulates the apple response to *B. dothidea*, provide new insights into plant–fungus interactions, and indicate the application potential of carbohydrate metabolism in the genetic breeding and cultivation of disease-resistant apple varieties.

## 4. Materials and Methods

### 4.1. Plant Materials, Cultivation, and Treatment

‘Orin’ apple calli were subcultured under basic growth conditions of 24 ± 0.5 °C and 24 h of darkness (at a relative humidity of 60–75%). The ‘Orin’ calli were subcultured in MS culture medium (1.5 mg·L^−1^ 2,4-D, 0.4 mg·L^−1^ 6-BA, 30 g·L^−1^ sucrose, and 7.5 g·L^−1^ agar; pH 5.8–6.0; autoclaved at 116 °C for 30 min). *B. dothidea* (preserved in our lab) was incubated on potato dextrose agar (PDA) medium at 28 ± 0.5 °C in darkness.

‘Golden Delicious’, ‘Fuji Nagafu No. 2’, and their segregating F1 hybrid population were planted in the Tai’an Tianping Lake base of the Shandong Institute of Pomology (117°032 E, 36°225 N) using routine management methods.

The pathogen infection experiment was performed according to the methods of He et al. [18]. The calli of the different lines were transferred to MS solid culture medium. Ten-day-old control and transgenic calli were infected by using 0.5-cm-diameter agar discs containing uniform *B. dothidea* mycelia, and then cocultured for 4 days in the dark. For the apple fruit infection experiments, fungi with strong and consistent mycelial growth were used. Then, a sterilized toothpick was inserted into a hole with a depth of 5 mm in the apple fruit; the fruit was filled with mycelia and incubated for 4 days. The treated calli and apple samples were collected, frozen in liquid nitrogen, and stored at −70 °C for subsequent experiments. Each experimental treatment was repeated at least three times.

### 4.2. Gene Cloning, Vector Construction, and Virus-Induced Gene Silencing (VIGS)

The open reading frames (ORFs) of *MdSYP121* were cloned from apple via PCR and inserted into a pPZP211-flag vector under the control of the cauliflower mosaic virus (CaMV) 35S promoter. The recombined constructs were transformed into *Agrobacterium tumefaciens* LBA4404. The transgenic apple calli were obtained as described by He et al. [18,44]. *MdSYP121*-TRV2 for VIGS was obtained by cloning selected fragments from *MdSYP121* into a pTRV2 vector. All primers used here are listed in Table S12. Then, the recombinant plasmid was transformed into *A. tumefaciens* strain GV3101, and a pTRV1 vector was also transformed into GV3101. The *A. tumefaciens* cells containing pTRV2 (or the recombinant plasmids) or pTRV1 were cultured and prepared as cell suspensions based on the description reported by Wang et al. [45]. The two types of cell suspensions (containing pTRV2 (or the recombinant plasmids) or pTRV1) were mixed in equal amounts and injected into the apple skin. The apple fruit infection experiment was the same as described above. The apple fruits were placed in a climate chamber (GreenFuture Co., Ltd. (Shanghai, China)) (temperature: 22 °C, photon flux density: 70 μmol·m^−2^·s^−1^). Apple fruit peels around the injection sites were collected for sugar content determination and gene expression analysis.

### 4.3. Metabolome Analysis

Three biological replicates were included for each group for metabolome sequencing. Metabolite extraction was performed according to the manufacturer’s instructions (METWARE Co., Ltd. (Wuhan, China)). The sample extracts were injected into a UPLC–ESI–MS/MS system for analysis. The UPLC–ESI–MS/MS analyses were performed using a ultra performance liquid chromatography (UPLC) system and a tandem mass spectrometry (MS/MS) system to the manufacturer’s instructions (METWARE Co., Ltd. (Wuhan, China)).

As a preliminary visualization of differences among different groups of samples, unsupervised principal component analysis (PCA) was performed using the statistics function prcomp within R according to the manufacturer’s instructions (METWARE Co., Ltd. (Wuhan, China)). Both hierarchical cluster analysis (HCA) and Pearson correlation coefficient (PCC) determination were carried out using the R package ComplexHeatmap [46]. The HCA results are presented as heatmaps with dendrograms. PCCs were calculated using the cor function in R and are presented only as heatmaps. The closer the correlation coefficient is to 1, the higher the similarity of metabolic abundance. Correlations were considered significant at *p* ≤ 0.05. For two-group analysis, differentially abundant metabolites were determined by the VIP (VIP ≥ 1) and absolute Log2FC (|Log2FC| ≥ 1.0). The identified metabolites were annotated using the KEGG Compound database and then mapped to the KEGG Pathway database [47]. The relative levels of all different metabolites identified using the screening criteria in all comparison groups were treated with unit variance (UV) scaling, and then a K-means cluster analysis was conducted.

### 4.4. Transcriptome Analysis

There were 3 biological replicates for each group for transcriptome sequencing. Each biological replicate contained ten independent calli from the OE and Vec lines. The extraction of total RNA was performed according to the kit manufacturer’s instructions. Both the total composite RNA from the OE lines and the total composite RNA from the Vec lines were used for Illumina sequencing at Novogene Co., Ltd. (Beijing, China). All procedures for cDNA library construction were performed according to a standard Illumina sample preparation protocol. The RNA-Seq libraries were sequenced on an Illumina Genome HiSeq platform.

Clean data (clean reads) were obtained by removing reads containing adapters, poly-N sequences, or low-quality reads from the raw data. At the same time, the Q20, Q30, and GC levels were calculated. The reference genome (GDDH13 Version 1.1) and gene model annotation files were downloaded from the genome website. The index of the reference genome was built using HISAT2. Fragments per kilobase of transcript sequence per million base pairs sequenced (FPKM) values of each gene was calculated based on the length of the gene and the number of reads mapped to the gene. A differential expression analysis of the two groups was performed using DESeq2. Gene Ontology (GO) and the Kyoto Encyclopedia of Genes and Genomes (KEGG) were used for the enrichment analysis of differentially expressed genes [48,49].

### 4.5. RNA Extraction and RT–qPCR Assays

RNA was extracted using an RNAprep Pure Plant Kit (TIANGEN, Beijing, China) in accordance with the manufacturer’s instructions. Then, a First Strand cDNA Synthesis Kit (Thermo Fisher Scientific, Shanghai, China) was used to synthesize first-strand cDNA. RT–qPCR was performed on the CFX96TM Real-time Detection System. The operational procedure was as described by He et al. [44]. The *Malus* × *domestica* actin gene (*MdActin*, GenBank ID XM029089583.1) was used as the housekeeping control. The primers used for RT–qPCR are shown in Table S12.

### 4.6. Measurement of Soluble Sugar, Glucose, Sucrose, Maltose, and Galactose Levels

The soluble sugars (the reducing sugars dissolved in water) were measured via anthrone colorimetry using a Plant Soluble Sugar Content Assay Kit (Solarbio, Beijing, China) according to the manufacturer’s instructions. The levels of glucose, sucrose, maltose, and galactose were determined using an HPLC system according to a previous report [50].

### 4.7. Statistical Analysis

The experiments were performed with at least three independent replicates. The variability of the samples is indicated by the mean ± standard error of three repetitions. Tukey’s HSD test in SPSS statistics software version 19 was used to determine statistical significance between different measurements.

## Data Availability

Data are contained within the article and Supplementary Materials.

## Supplementary Materials

The supporting information can be downloaded at: https://www.mdpi.com/article/10.3390/ijms242216242/s1.
