# Supplementary material for: Metabolome and Transcriptome Profiling Reveals the Function of MdSYP121 in the Apple Response to Botryosphaeria dothidea
Source: Int J Mol Sci. 2023 Nov 13;24(22):16242. doi: 10.3390/ijms242216242 (PMC10671699; doi:10.3390/ijms242216242)
Supplement: Supplementary file 1 [file ijms-24-16242-s001.zip › supplementary Figures S1-S2.pdf]

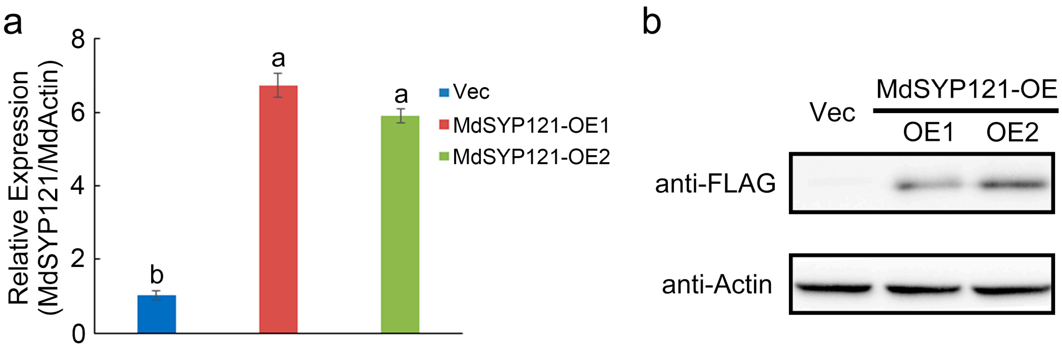

Figure S1. Representative phenotypes of transgenic apple plants and identification of *MdSYP121*-OE transgenic apple calli. (a) *MdSYP121*-OE transgenic apple calli were confirmed by RT-qPCR and Western blot RT-qPCR and Western blot. (b) Representative phenotypes of Vec and *MdSYP121*-OE calli lines with *B. dothidea* infection for 4 days.

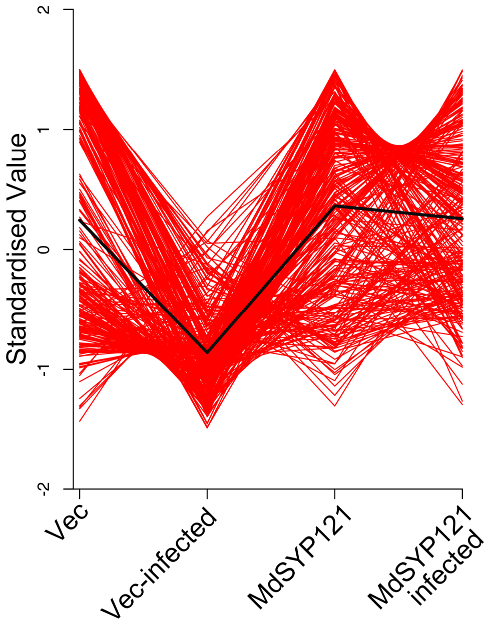

Figure S2. K-Means clustering analysis in *MdSYP121*-OE and Vec calli infected with *B. dothidea*.
